# Vitamins and Radioprotective Effect: A Review

**DOI:** 10.3390/antiox12030611

**Published:** 2023-03-01

**Authors:** Inés Lledó, Blanca Ibáñez, Ana Melero, Alegría Montoro, Juan F. Merino-Torres, Nadia San Onofre, Jose M. Soriano

**Affiliations:** 1 Food & Health Lab, Institute of Materials Science, University of Valencia, 46980 Paterna, Spain; 2 Department of Pharmacy and Pharmaceutical Technology and Parasitology, Faculty of Pharmacy, University of Valencia, 46100 Burjassot, Spain; 3 Service of Radiological Protection, Clinical Area of Medical Image, University and Polytechnic La Fe Hospital, 46026 Valencia, Spain; 4 Biomedical Imaging Research Group GIBI230, Health Research Institute (IISLaFe), University and Polytechnic La Fe Hospital, 46026 Valencia, Spain; 5 Joint Research Unit on Endocrinology, Nutrition and Clinical Dietetics, University of Valencia-Health Research Institute La Fe, 46026 Valencia, Spain; 6 Department of Endocrinology and Nutrition, University and Polytechnic La Fe Hospital, 46026 Valencia, Spain; 7 Department of Medicine, Faculty of Medicine, University of Valencia, 46010 Valencia, Spain; 8 Department of Community Nursing, Preventive Medicine and Public Health and History of Science, University of Alicante, 03690 Alicante, Spain

**Keywords:** radioprotection, vitamins, antioxidants, radiation

## Abstract

The radioprotective effect ex vivo, in vitro and in vivo of vitamins was reviewed using PubMed and Embase and conducted according to the PRISMA statement. A total of 38 articles were included in this review, which includes the radioprotective effect of vitamins from ex vivo, in vitro and in vivo studies. Vitamins A, C, D and E were used alone, in combination or with other nutritional and non-nutritional compounds. The use of vitamins in natural form or supplementation can be useful to reduce the radiation effect in the body, organs and/or cells. Only four (A, C, D and E) out of thirteen vitamins have been detected with radioprotective properties being mainly vitamin E followed by vitamin C, A and D.

## 1. Introduction

Radiation is energy travelling as waves or particles and is part of our everyday environment [1]. There are two types of radiation [2] according to the effects they produce in the matter: (i) ionizing radiation, which is an energy form that is able to ionize atoms, that is, removing electrons from the atoms. Including as ionizing radiation is α- and β-radiations, neutron particles, γ-radiation and X- rays. (ii) Non-ionizing radiation, a form of radiation with less energy than ionizing radiation. It includes electric and magnetic fields, radio waves, microwaves and optical radiation, which consists of infrared, visible and ultraviolet radiation. This second type is not able to cause ionization. The penetration capacity of ionizing radiation and its radiation energy can be absorbed by living tissues causing harmful effects to living organisms, especially the most penetrating ones and the at high levels of exposure [3]. Radiation exposure is the amount of X-ray or γ-radiation that interacts in a volume of air. The units of measurement for radiation exposure are Roentgen (R, traditional U.S. unit) or Coulomb/kilogram (C/kg, international unit), while radiation dose is the quantity of absorbed energy per unit mass with the gray (Gy) as the unit of international measure [4].

During ionizing radiation exposure, the generated reactive oxygen species (ROS) result in cellular damage. Free radicals induce DNA damage that can have terrible effects, such as gene alteration, cell death and genomic instability, among others. Most of this radiation damage comes from the interaction of ionizing radiation with biological molecules to produce free radicals; therefore, the compounds that can reduce or delete free radicals or prevent formation of these radicals can negate these mechanisms and function as radioprotectors. There are different types of substances that reduce the lethal effect of radiation: radioprotectors (including sulfhydryl compounds and antioxidants), adaptogens (stimulate radio resistance) and absorbents (prevent incorporation of iodine). They are used in clinical practice and are mainly based on their mechanism of action in the suppression of the formation of reactive species and detoxification of radiation-induced species. An ideal radioprotective agent should prevent direct acute or chronic effects on normal tissue, be easily dispensed without toxicity and not protect tumors from irradiation [5]. Nowadays, these compounds, including vitamins, are applied in space travel, personnel handling nuclear emergencies and individuals subjected to diagnostic and therapeutic radiation exposures due to their ability to reduce the effects of breaks (including single-strand and double-strand; DSB), base damage and DNA–protein cross-links.

Vitamins are organic compounds different from fats, carbohydrates and proteins and are classified as nutrients, which are present in foods that are essential for normal physiological function (i.e., maintenance, growth, development and/or production) and preventing specific deficiency syndromes, which occurs when the vitamin is absent, underutilized or is not synthesized by the host in amounts adequate to meet normal physiological needs. They are chemically very heterogeneous and have been classified into two major groups according to solubility: (i) liposoluble are soluble in lipids but not in water and are, therefore, usually in the fat of the food (vitamins A, D, E and K). These can accumulate and cause toxicity when ingest in large quantities. (ii) Water-soluble, including vitamins B1, B2, niacin, pantothenic acid, B6, biotin, folic acid, B12 and vitamin C [6].

The aim of this review was to evaluate the radioprotective effect ex vivo, in vitro and in vivo of vitamins in single us, combination form, or with other nutritional and non-nutritional compounds.

## 2. Materials and Methods

**Figure 1:**
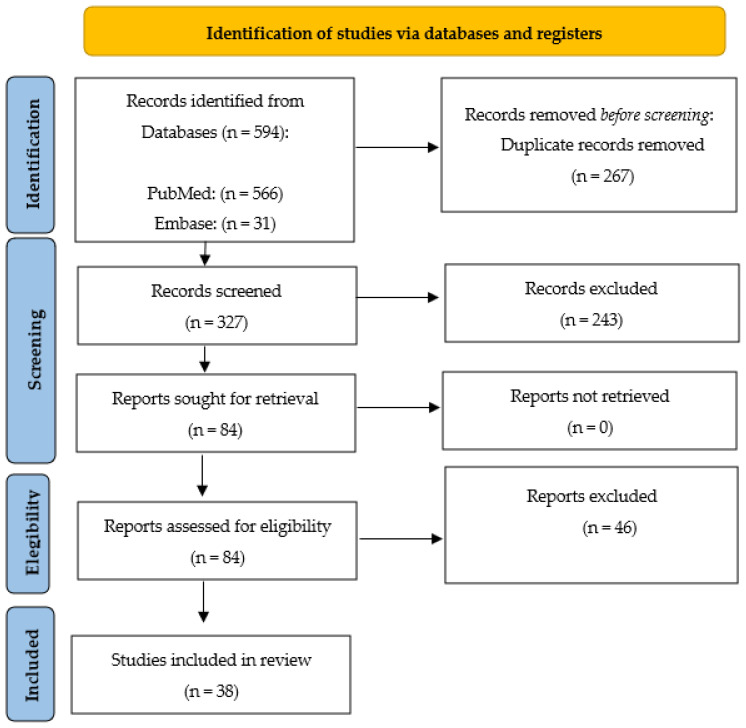
PRISMA (Preferred Reporting Items for Systematic Reviews and Meta-Analyses) flow diagram [7].

We carried out this review using the Preferred Reporting Items for Systematic Reviews and Meta-Analyses (PRISMA) statement [7] (Figure 1) using PubMed and Embase databases.

The PubMed search was conducted using the medical subject headings (MeSH) terms: ‘vitamin’, ‘radioprotective’ and ‘radiation’. The terms ‘vitamin’, ‘radioprotector’, ‘radioprotective’ and ‘radiation’ were searched in Embase. Boolean operators AND or OR were utilized during combinatory keywords search. In both databases, the search was limited to English language published from January 2001 to January 2023.

The inclusion criteria were ex vivo, in vitro and in vivo studies from original papers, review papers, theses and experimental procedures. The exclusion criteria included only abstracts, manuscripts with unrelated abstracts, books, letters, conference literature, case reports, editorials and pilot studies. Full-text articles of all shortlisted abstracts were then screened for eligibility.

Three teams of paired reviewers (I.L., B.I.; A.M., N.S.O.; J.-F.M.-T., J.M.S.) with expertise in medical and health assessment and training in research methodology independently screened three sections (titles, abstracts and full texts if eligible), assessed generalizability and collected data in reviews. Any disagreements were resolved by other researcher (A.Mo.).

## 3. Results and Discussion

Table 1, Table 2 and Table 3 show the radioprotective effect of the vitamins from ex vivo, in vitro and in vivo studies, respectively. Results reflected that most research (*n* = 27) was with in vivo studies followed by in vitro (*n* = 9) and ex vivo (*n* = 2).

Reviewed studies reflected that only four vitamins have potential radioprotective effects being ranked from highest to lowest: vitamin E, C, A and D.

The characteristic structure of vitamin E contains a phenolic ring adjacent to a heterocyclic ring containing oxygen. It has eight different isoforms: four are saturated analogues (α, β, γ and δ) under the name of tocopherols, and the other four are unsaturated analogues called tocotrienols. The difference between the tocopherols is found in the methyl groups of the phenolic ring, with these differences being the ones determine the condition of vitaminic and antioxidant activity (which is inverse to the vitaminic activity). They are antioxidants because they can yield a hydrogen radical to the environment and paralyze the progression of free radicals. The first studies on these properties in vitamin E were carried out in 1970s. Malick et al. [46] administered an aqueous preparation of α-tocopherol intraperitoneally in experimental animals, and one year later, Konings [47] conducted an in vivo study, which demonstrated the protective role of α-tocopherol against oxidative damage and lipid peroxidation induced by ionizing radiation in mice ( administered as an aqueous preparation of α-tocopherol before radiation). Vitamin E has been shown to have a protective role at the level of the ileum and colon in rats that had received different doses of ionizing radiation [48], thus improving absorption in these sections of the gastrointestinal system. Additionally. Vitamin E has been shown to improve survival of irradiated mice [49]. Paranich et al. [50] found that the radioprotective effect of the potassium form of α-tocopherol phosphate was superior to other forms studied so far. In 1995, a derivate of vitamin E, TROLOX, reduced in vitro radiation-induced apoptosis in MOLT-4 cell lines when administered after exposure [51]. Additionally, another in vitro study showed that radioprotective substances could be effective in controlling tumour growth without exerting any protective effect on cancer cells [52]. Lefaix [53] used vitamin E to investigate its role in fibrotic processes, demonstrating that the protective capacity of vitamin E together with other drugs could improve such processes commonly produced in different systems after exposure to this type of radiation. According to reviewed ex vivo and in vitro studies, this vitamin is assessed in single use studies as γ -tocotrienol [9] and dl-α-tocopherol [12], respectively, being useful for the maintenance of intestinal permeability and structure and to help reduce DNA from human lymphocytes, respectively. However, in vivo studies of vitamin E [21,22,23,24,25,26] as the most common isoform in human and animal tissues, have been displaced by other forms, such as γ- [27,28] and δ- [29,30,31,32,33] tocotrienol, which appear better than other tocols as radiation protectors and radiation mitigators.

On the other hand, γ-tocopherol-N, N-dimethylglycine ester, which is a type of water-soluble vitamin E derivative, acts as a prodrug of γ-tocopherol, but it is interesting that its major metabolite is 2,7,8-trimethyl-2S-(beta-carboxyethyl)-6-hydroxylchroman [34]. The latter compound contributes to the radiation ameliorating effect following accidental overexposure. Furthermore, Mutalip [54] reflected that better antioxidant properties were observed in tocotrienols compared to tocopherols. However, the literature reflected that tocotrienols showed a higher radioprotective effect in comparison with tocopherols, remembering that low bioavailability is a limiting factor in a clinical viewpoint when applied as radioprotectants [55]. On the other hand, Singh and Hauer-Jensen [56] indicated that 75 mg/kg of α-tocopherol caused negative effects or death in non-human primates due to the use of vitamin E and its derivatives. While this dose may have enhanced radioprotective activity, it may be toxic to humans. However, in the following years =, there were several studies (Table 3) that confirm the radioprotective effect of vitamin E. Suhardi et al. [57] showed the biocompatible free radical scavenger vitamin E minimizes the adverse effects of gamma sterilization (performed at 25 kGy irradiation), especially in a bone allograft.

Vitamin C, a water-soluble vitamin, could be useful for its antioxidant activity and pro-oxidative factor [58]. Its antioxidant activity originates from a double bond to two alcohol groups since alcohol groups can easily oxidize from an alcohol to a ketone by destroying the existing double bond. When this occurs, it is called dehydroascorbic acid, and it is a reaction that is in a reversible balance. Dehydroascorbic acid can also oxidize to L-gulonic acid in an irreversible reaction; however, L-gulonic acid has no vitaminic activity. Due to its ability to oxidize and protect other molecules, vitamin C is unstable in the presence of oxygen. This nutrient protects mice from the lethality of the effects of radiation and skin peeling. Mice transplanted with fibrosarcoma were orally (4.5 g/kg.c) administered 50 min before full body irradiation [59]. There was a decrease in chromosomal and micronucleus aberrations in bone marrow cells provided by oral vitamin C pre- and post-irradiation administration [60]. Given as a skin injection and ingestion through the diet, vitamin C has the ability to reduce radio-induced damage, considering spermatogenesis and sperm survival as a biological target [61]. An in vitro study performed by Cai et al. [17] demonstrated that thymus cells of experimental animals were treated with this vitamin showing significantly reduced DNA damage induced by gamma radiation (between 30 and 50%) showing a protective effect similar to that of glutathione. Additionally, its use in differentiated thyroid cancer patients ablated with radioiodine demonstrated that it ameliorated serum oxidative stress [19].

Different forms of this vitamin are ascorbic acid 2-glucoside, 6-palmitoyl ascorbic acid-2-glucoside (PAsAG) [8] and 6-o-palmitoylascorbate (PlmtVC) [11], which have been useful in ex vivo and in vitro studies, respectively, due to their susceptibility to thermal and oxidative degradation. The first compound can protect the hemopoietic system from radiation-induced lesions. The recommendation is the administration of 80 mg/kg b.w. one hour prior to whole body 6 Gy gamma radiation, which protected cellular DNA in spleenocytes, bone marrow cells and blood leucocytes from radiation damage [8]. PlmtVC is a lipophilic vitamin C derivative with interesting properties having an adequate molecular hydrophilicity–lipophilicity balance [11]. In fact, oral administration of this compound favors the separation of palmitic acid by hydrolysis due to cellular esterases in the stomach, converting it to the active antioxidant form (ascorbic acid) in the distribution to tissue stores [62] and keeping a high level of intracellular vitamin C, resulting in relief to X-ray-induced oxidative stress [11].

Neither vitamin A nor D have been used ex vivo, and no in vitro studies verify their radioprotective properties. Single use vitamin A was not utilized; however, it was for vitamin D [20], which demonstrated anti-inflammatory, antioxidant and radioprotective effects on lacrimal glands in histopathologic and tissue cytokine and oxidant/antioxidant level evaluations in rats. On the other hand, four reviewed vitamins were used in combination (vitamins C and E and β-carotene [15]; vitamins E and C [35]; vitamin A, C and E [36]; and vitamins A, E, C and over-the-counter multivitamins [37]) or other nutritional (selenium [16]; copper [17]; and magnesium sulphate [38]) and non-nutritional (Haberlea rhodopensis extract [18]; β-D-glucan [39]; curcumin [40]; famotidine and cimetidine [41]; famotidine [42]; melatonin [43]; amifostine and L-carnitine [44]; and Nigella sativa oil and melatonin [45]) compounds. The highest protection factor value (4.3) was obtained with vitamin C and famotidine helping to reduce the frequency of micronucleous polychromatic erythrocytes after irradiation [42]. Synergistic or antagonistic effects for the combined use of vitamins were shown, which may at times depend on several factors, including the cell or model type and the physiological context (highly relevant to the question of radioprotective properties).

For future directions, it is very important to think that ionizing radiation has different and important applications. However, the harmful biological effects induced by the exposure of normal body tissue to ionizing radiation are one of its most important limitations. For this reason, a risk–benefit balance is required for the patient when ionizing radiation is applied in environments or medical applications. Analyzing the amount of research in vitamin radioprotection, there has been a big increase in the last 40 years, and it is clear that the application of radiation biology has gained greater relevance and significance in health and environmental issues. On the one hand, nuclear terrorism and weapon related effects are raising alarms and concerns for public health. On the other hand, radiation biology research has great potential in diagnosis, therapy and establishing standards for assessment risk from radiation exposure. The development of effective medical countermeasures against radiation is of immense importance to our future health and welfare. Furthermore, if humanity wants to explore space, there is a need to effectively protect people against cosmic radiation. In spite of this, there are several questions that need to be answered, such as the fact that radiation injury mechanisms are not yet completely identified, the potential in vivo toxicity associated with agents under development, how long a radioprotector or radiomitigator will work after radiation exposure, why some radioprotectors selectively target normal but not cancer cells, the fact that some radioprotectors also show anticancer properties and, finally, the market size for these compounds relative to the investment required. Although the truth is that more in vivo research is needed to determine the radioprotective effect of vitamins that is seen in various studies is really effective and useful in clinical practice, there are potential pharmacological agents with different targets and mechanisms to act as radioprotectors that are subject to further research to prevent, alleviate or treat ionizing radiation-induced toxicity.

## 4. Conclusions

The use of vitamins in their natural form or supplementation can be useful to reduce radiation effects in the body, organs and/or cells. Only four (A, C, D and E) out of thirteen vitamins have been detected with radioprotective properties, namely vitamin E followed by vitamin C, A and D. However, different variables were observed in these studies, including type of assay (ex vivo, in vitro and in vivo), administrated radiation type and dose, studied sample (mice, cells and patients), concentration of vitamin/s and single use, combination form or with other nutritional and non-nutritional compounds, among others. Furthermore, the balance of low concentrations of vitamins and the high radioprotective properties to reduce some of their harmful effects is interesting. More work at the cellular, molecular and whole animal or human level of vitamins is needed to guarantee the security and efficiency against radiation effects.

## Figures and Tables

**Table 1:** Ex vivo studies about radioprotective effect of vitamins.

Compound/s	Radiation Type and Dose	Studied Sample	Main Outcomes	Reference
6-palmitoyl ascorbic acid-2-glucoside (PAsAG)	^60^Co γ -irradiation (6 Gy)	Mouse spleen cells	Cellular DNA protected due to the presence of 1.6 mM PAsAG during radiation so that the comet parameters.	Chandrasekharan et al., 2009 [8]
γ -tocotrienol (GT3)	^137^Cs (8 Gy to measure apoptosis of epithelial cells and recovery of the immune cells and 10 and 12 Gy for the intestinal permeability assay)	Male CD2F1 mice	Cell death and decrease in villus height were suppressed and decreased, respectively, together with other intestinal effects, such as are crypt depth, attenuated intestinal permeability, and upregulated occludin level in the intestine, due to the use of GT3 (200 mg/kg b.w.) administered 24 h before irradiation.	Garg et al., 2019 [9]

**Table 2:** In vitro studies about radioprotective effect of vitamins.

Compound/s	Radiation Type and Dose	Studied Sample	Main Outcomes	Reference
Vitamin D	X-rays (4 Gy)	Human umbilical vein endothelial cells (HUVECs)	It was shown that vit D reduced IR-induced reactive oxygen species production protecting proliferating and quiescent HUVECs from cellular apoptosis or senescence, respectively, by regulating positively the mitogen-activated protein kinase (MAPK) pathways.	Marampon et al., 2016 [10]
Vitamin C (6-o-palmitoylascorbate (PlmtVC))	X-rays (1.5 Gy)	HEV0082 cells	PlmtVC markedly inhibited X-ray induced caspase 3 activation. So, PlmtVC could prevent X-ray-induced DNA damages and inhibited intracellular ROS production and lipid peroxidation.	Xiao et al., 2014 [11]
Vitamin E (as dl-α-tocopherol).	^60^Co γ-irradiation (6 Gy)	Human lymphocytes	Reduction of DNA damage up to 94.2% using concentration of 0.8 mM of vitamin E.	Darlina et al., 2017 [12]
Vitamins C and E	^60^Co γ-irradiation (0.1–3 Gy)	Bovine serum albumin (BSA)	Irradiated BSA, with the presence of both vitamins, was protected of structural changes caused by ROS.	Zarei et al., 2021 [13]
Vitamins E and C	^131^I (20 µCi)	Human lymphocytes	Vitamins E and C can reduce the toxicity of ^131^I. Vitamin C provided the more protection for DNA followed of vitamin E.	Safaei et al., 2018 [14]
Vitamins C and E and β-carotene	^60^Co γ-irradiation (2 Gy)	Human lymphocytes	The strongest effect is more effective when they were added no later than 1 h after irradiation. Furthermore, vitamin C at low concentration (1 μg/mL), vitamin E at the concentration above 2 μg/mL and β-carotene were effective at all tested concentrations between 1–5 μg/mL reduced the number of micronuclei in irradiated cells.	Konopacka et al., 2001 [15]
Vitamin E and selenium	X-rays (2 Gy)	Human lymphocytes	1 hr after administration of both nutrients was observed the maximum protection and decrease in frequency of micronuclei (50%).	Rostami et al., 2016 [16]
Vitamin C and copper	^60^Co γ-irradiation (3 Gy)	Calf thymus DNA	Vitamin C with copper (50 μM) significantly enhanced γ-radiation-induced DNA damage.	Cai et al., 2001 [17]
Vitamin C and Haberlea rhodopensis extract	^60^Co γ-irradiation (2 Gy)	Rabbits peripheral blood lymphocytes	The frequency of dicentrics and double acentric fragments was similar with both compounds.	Popov et al., 2010 [18]

**Table 3:** In vivo studies about radioprotective effect of vitamins.

Vitamin/s	Radiation Type and Dose	Studied Sample	Main Outcomes	Reference
Vitamin C	^131^I (5550 MBq)	Differentiated thyroid cancer patients ablated with radioiodine	This vitamin, used after radioiodine therapy, ameliorated serum oxidative stress.	Jafari et al., 2018 [19]
Vitamin D	^131^I (3 mCi)	Wistar albino rats	Anti-inflammatory, antioxidant and radio-protective effects on lacrimal glands were observed.	Eksioglu et al., 2019 [20]
Vitamin E	^60^Co γ-irradiation (15 Gy)	Male Wistar rats between 8 and 10 weeks old divided into six groups.	Vitamin E protected the salivary function 30 days after irradiation.	De Moraes ramos et al., 2006 [21]
Vitamin E	^60^Co γ-irradiation (0.6 Gy/min)	Male CD2F1 mice	Vitamin E at a dose of 400 IU/kg acts as radioprotectant against lethal doses of radiation being more efficacious when is given subcutaneously than when given orally.	Sree et al., 2002 [22]
Vitamin E	^131^I (100 mCi)	Eighty-two patients with differentiated thyroid cancer	Vitamin E exerts significant protective effects on the parotid and submandibular glands after ^131^I therapy.	Upadhyaya et al., 2017 [23]
Vitamin E	^60^Co γ-irradiation (15 Gy)	Sixty male Wistar rats	Vitamin E was not effective as a radioprotective agent on acinar cells in rats’ parotid glands.	Gomes et al., 2013 [24]
Vitamin E	^60^Co γ-irradiation (15 Gy)	Male Wistar rats	Vitamin E at dose 400 IU/Kg significantly protected and improved salivary gland function against toxicity induced by ionizing radiation.	Abedi et al., 2015 [25]
Vitamin E	^131^I (3 mCi)	Wistar albino rats	Histopathological examinations revealed that vitamin E protects rat lacrimal glands against radioiodine-related early damage.	Acar et al., 2013 [26]
γ-tocotrienol	^60^Co γ-irradiation (9.2 Gy)	Male CD2F1 mice	Induction of high levels of granulocyte colony-stimulating factor by γ-tocotrienol administration is responsible for its protective efficacy against radiation injury.	Kulkarni et al., 2013 [27]
γ-tocotrienol	^60^Co γ-irradiation (12 Gy)	Rhesus macaques were treated with 37.5 mg/kg γ-tocotrienol subcutaneously 24 h prior to radiation exposure	γ-tocotrienol has radioprotective function in intestinal epithelial and crypt cells.	Garg et al., 2022 [28]
δ-tocotrienol	^60^Co γ-irradiation (0, 5 or 8.75 Gy)	CD2F1 male mice	δ-tocotrienol protects mouse bone marrow from radiation-induced damage through extracellular signal-related kinase activation-associated mammalian target of rapamycin survival pathways.	Li et al., 2010 [29]
δ-tocotrienol	^60^Co γ-irradiation (7 Gy)	Mouse liver microsomes	24 h prior to 7 Gy reduced pancytopenia significantly with 300 mg/kg δ-tocotrienol.	Satyamitra et al., 2011 [30]
δ-tocotrienol	^60^Co γ-irradiation (0.5, 11 or 11.5 Gy)	Male CD2F1 mice	200 mg/kg subcutaneously of this vitamin 24 h before irradiation in mice obtained a dose reduction factor of 1.29 and accelerated the recovery of several parameters, such as total white blood cells, neutrophils, monocytes, platelet, and reticulocytes.	Ghosh et al., 2009 [31]
δ-tocotrienol	^60^Co γ-irradiation (0 or 7 Gy)	Male CD2F1 mice	This vitamin increased serum levels of G-CSF, IL-6, KC and several other cytokines within 12–24 h post-administration.	Kulkarni et al., 2012 [32]
δ-tocotrienol	^60^Co γ-irradiation (9.2 Gy)	Male CD2F1 mice	Granulocyte colony-stimulating factor induced by δ-tocotrienol administration a protective efficacy against radiation injury.	Singh et al., 2014 [33]
γ-tocopherol- N, N-dimethyl glycine ester (GTDMG)	X-rays (7.5 Gy)	Male C3H mice	GTDMG enhanced the recovery of red and white blood cells, platelet counts and significantly increased the number of endogenous spleen colonies.	Anzai et al., 2014 [34]
Vitamin E and vitamin C	^137^Cs γ-irradiation (2 or 8 Gy)	Wistar male rats	The pretreatment with both vitamins provided radioprotection partially by aiding non-inflammatory, apoptotic elimination of several damaged cells.	Vasilyeva et al., 2015 [35]
Vitamin A, C, E and lycopene	^60^Co γ-irradiation (8 Gy)	Swiss Albino rats were divided into six groups.	Vitamin E supplementation, compared to other vitamins, was most potent in ameliorating the intestinal aberrations.	Anwar et al., 2013 [36]
Vitamins A, E, C and over-the-counter multivitamins	^60^Co γ-irradiation (8.8 Gy)	Male Balb/c mice	The radioprotective effect of vitamin C is more efficient than the effect of other vitamins. Even high doses of vitamin C can show lifesaving radioprotective effects.	Mortazavi et al., 2015 [37]
Vitamin A and magnesium sulphate	X-rays (2 Gy)	Mice were treated intraperitoneally with 9 different combined doses of vitamin A (100, 200 and 400 mg/kg) and MgSO_4_ (75, 150 and 300 mg/kg)	Combination of 200 mg/kg vitamin A + 150 mg/kg MgSO_4_ produced high protection against 2Gy X-ray protecting the bone marrow cells of mice.	Mirdoraghi et al., 2022 [38]
Vitamin E and β-D-glucan	^60^Co γ-irradiation (6, 7 or 8 Gy)	240 female mice	Β-D-glucan in the body of mice, during exposure to ionizing radiation, leads to dose reduction factor higher than one. Furthermore, both increased resistance of mice against ionizing radiation.	Tabeie et al., 2017 [39]
Vitamin C and curcumin	γ-radiation (0, 3 or 6 6 Gy)	Human non-smoking male volunteers were treated with orally given 100 µg/mL of vitamin or curcumin before the radiation	The extent of DNA damage was significantly decreased either in the presence or following the intake of curcumin and ascorbic acid. The intake of dietary antioxidants such as ascorbic acid could offer protection against ionizing radiation reflected, in peripheral blood leukocytes, cellular DNA damage.	Nair and Menon, 2013 [40]
Vitamin C, famotidine and cimetidine	^60^Co γ-irradiation (2 Gy)	Male NMRI mice	Oral administration of Famotidine, vitamin C and Cimetidine, in single use or combination form, demonstrates reliable and similar radioprotective effects.	Naeeji et al., 2017 [41]
Vitamin C and famotidine	^60^Co γ-irradiation (2 or 4 Gy)	Male NMRI mice	Combination of both compounds was more effective in reducing the frequency of micronucleated polychromatic erythrocytes leading to a protection factor of 4.3 after irradiation.	Zangeneh et al., 2015 [42]
Vitamin C and melatonin	X-rays (7.5 Gy)	Volunteers	Before irradiation, in human peripheral blood lymphocytes, the use of both compounds caused a reduction in DNA damage.	Aram et al., 2016 [43]
Vitamin E, amifostine and L-Carnitine	^131^I (555–660 MBq)	Adult guinea pigs	The individual use of these compounds for radioprotection yields different levels of, but not absolute, protection against radioactive iodine treatment injury in salivary glands.	Torun et al., 2019 [44]
Vitamin E, Nigella sativa oil and melatonin	X-ray (8 Gy)	Albino rats	The use of these compounds could limit radiation induced injury in brain and cerebellum.	Nor-Eldin and Elsayed, 2019 [45]
